# The predictive utility of the plant phylogeny in identifying sources of cardiovascular drugs

**DOI:** 10.1080/13880209.2018.1444642

**Published:** 2018-02-27

**Authors:** Emily Guzman, Jeanmaire Molina

**Affiliations:** Department of Biology, Long Island University, Brooklyn, NY, USA

**Keywords:** Drug discovery, ethnobotany, ethnomedicine, evolutionary pharmacology, natural products

## Abstract

**Context:** Cardiovascular disease (CVD) is the number one cause of death globally, responsible for over 17 million (31%) deaths in the world. Novel pharmacological interventions may be needed given the high prevalence of CVD.

**Objective:** In this study, we aimed to find potential new sources of cardiovascular (CV) drugs from phylogenetic and pharmacological analyses of plant species that have experimental and traditional CV applications in the literature.

**Materials and methods:** We reconstructed the molecular phylogeny of these plant species and mapped their pharmacological mechanisms of action on the phylogeny.

**Results:** Out of 139 plant species in 71 plant families, seven plant families with 45 species emerged as phylogenetically important exhibiting common CV mechanisms of action within the family, as would be expected given their common ancestry: Apiaceae, Brassicaceae, Fabaceae, Lamiaceae, Malvaceae, Rosaceae and Zingiberaceae. Apiaceae and Brassicaceae promoted diuresis and hypotension; Fabaceae and Lamiaceae had anticoagulant/thrombolytic effects; Apiaceae and Zingiberaceae were calcium channel blockers. Moreover, Apiaceae, Lamiaceae, Malvaceae, Rosaceae and Zingiberaceae species were found to possess anti-atherosclerotic properties.

**Discussion and conclusions:** The phylogeny identified certain plant families with disproportionately more species, highlighting their importance as sources of natural products for CV drug discovery. Though there were some species that did not show the same mechanism within the family, the phylogeny predicts that these species may contain undiscovered phytochemistry, and potentially, the same bioactivity. Evolutionary pharmacology, as applied here, may guide and expedite our efforts in discovering sources of new CV drugs.

## Introduction

Cardiovascular disease (CVD), a group of disorders affecting the heart and blood vessels, is the number one cause of death globally, responsible for 17.7 million deaths in 2015 (31%), mostly in low and middle-income countries (WHO 2017). In the USA, one out of every three deaths is attributed to CVD and is the leading cause of death, surpassing accidents and any other type of disease (Benjamin et al. 2017). Coronary heart disease and stroke, the deprivation and blockage of oxygen-rich blood to the heart and brain, respectively, make up 80% of these deaths. Blockage of the coronary and cerebral arteries is usually due to accumulation of fatty deposits within the blood vessel. However, strokes may also occur when the cerebral vessels burst or when there is a blood clot (Roth et al. 2017; WHO 2017). Yet, mortality from heart attack and strokes may be prevented by reducing risk factors including consumption of unhealthy diet, lack of physical activity, tobacco and alcohol use, as well as managing morbidities such as hypertension, hyperlipidaemia, atherosclerosis and diabetes with medication (Benjamin et al. 2017; WHO 2017). Novel pharmacological interventions may be needed given the high prevalence of CVD.

Traditional medicine has paved the way for the development of modern cardiovascular (CV) drugs including aspirin, digoxin, amiodarone and reserpine (Mashour et al. 1998; Fabricant and Farnsworth 2001; Li et al. 2015). Aspirin, currently used as an analgesic and anticoagulant, was developed by Bayer in the 1890s (Norn et al. 2009) from the natural product, salicin found in the bark of white willow, *Salix alba* L. (Salicaceae). However, its antithrombotic potential was not appreciated until 1950s. Aspirin therapy has been shown to significantly reduce vascular mortality by 23% (Almony et al. 1996). *Digitalis purpurea* L. (Plantaginaceae), commonly known as foxglove, is the source of the cardiac glycoside digoxin, which is prescribed for patients with congestive heart failure (Campbell and MacDonald 2003). William Withering, an 18th c. English physician, learned the use of foxglove from a folk herbalist, and determined its clinical effects particularly in treating dropsy (oedema) through its action of increasing the intensity of cardiac contractions (Krikler 1985). *Ammi visnaga* (L.) Lam. (Apiaceae), an ancient Egyptian medicinal plant, was found to have cardioactive properties resulting in the development of the anti-arrhythmia drug, amiodarone, in the 1960s from khellin, the plant’s active natural product (Bhagavathula et al. 2015). In India, the root of *Rauvolfia serpentina* (L.) Benth. ex Kurz (Apocynaceae) has long been used for psychosis and as a sedative (Mashour et al. 1998; Rätsch 2005). The indole alkaloid reserpine was isolated from the root, and since 1950s has been used to reduce blood pressure (Lobay 2015). These examples highlight the importance of traditional medicine in drug discovery, with 80% of 122 compounds used globally as drugs having ethnomedicinal origins (Fabricant and Farnsworth 2001).

Interestingly, ethnobotanically important plant species analysed in a phylogenetic context could reveal pharmacologically relevant plant families, with various species being used similarly by different cultures, a pattern of cultural convergence (Saslis-Lagoudakis et al. 2012; Alrashedy and Molina 2016; Xavier and Molina 2016; Molina 2018). These studies demonstrate that different cultures have independently discovered – yet converged on similar medicinal applications for species of the same family. This is strong evidence for the therapeutic potential of these related plants, that they possess phylogenetically conserved phytochemistry and pharmacology that may be experimentally explored.

In the present study, we aimed to identify potential new sources of CV drugs from phylogenetic and pharmacological analyses of plant species that have CV applications in the literature based on traditional and experimental evidence. We reconstructed the molecular phylogeny of these plants and map their pharmacological mechanisms of action to determine if there are common mechanisms within families, as would be expected due to common ancestry. This produced a phylogenetic scaffold that may guide CV drug discovery in related plant species that have not been tested experimentally.

## Materials and methods

PubMed was mined for plant species (total number = 139 species) that have experimental evidence of CV activity, as well as for plant species used ethnobotanically for CV applications (Table 1). Congeneric species were avoided so as not to bias the results on multispecies genera as a more comprehensive sampling of CV plant taxa was desired. Cardiovascular mechanisms of action for each plant species were classified according to Klabunde (2012), when it can be determined from the reference. The *rbcL* sequence for each plant species was obtained from GenBank following methods in Xavier and Molina (2016). These sequences were then aligned using the program MAFFT v.7 (Katoh and Standley 2013). The maximum likelihood phylogeny was reconstructed using PhyML applying the GTR substitution model and SH-like branch support (Guindon et al. 2010). The phylogeny was uploaded in ITOL (Interactive Tree of Life http://itol.embl.de/; Letunic and Bork 2016) where CV mechanisms of action were mapped on the phylogeny. These mechanisms included angiotensin-converting enzyme inhibitors (ACEI), α/β-adrenoceptor blockers (AB), calcium-channel blockers (CCBs), cardiac glycosides or Na^+^/K^+^ ATPase inhibitors (CG), diuretics (DIU), nitrodilators (NDs), phosphodiesterase-inhibitors (PDEIs), potassium channel blockers (PCBs), anticoagulants/thrombolytics (TL), endothelium-receptor antagonist (ERA), muscarinic receptor antagonist (MRA), venotonic (VENO) and anti-atherosclerosis (AA). MU was indicated when the mechanism is unknown. Plant families with at least four species, with majority of the species showing a common mechanism of action, were highlighted. The predominant mechanism of action for plant species with experimental evidence may be assumed as the potential mechanism for confamilial species that only have ethnobotanical/traditional use to date.

**Table 1.** Plant species with cardiovascular applications based on experimental or ethnobotanical/traditional evidence.

Plant species	Family	References	Evidence	Mechanism of action
*Andrographis paniculata* (Burm.f.) Nees	Acanthaceae	Liu and Huang (2016)	Experimental	ACEI
*Acorus calamus* L.	Acoraceae	Anwar et al. (2016)	Experimental	CCB
*Echinodorus grandiflorus* (Cham. & Schltdl.) Micheli	Alismataceae	Anwar et al. (2016)	Experimental	ND
*Achyranthes bidentata* Blume	Amaranthaceae	Xiong et al. (2013)	Traditional	DIU
*Chenopodium murale* L.	Amaranthaceae	Ibarra-Alvarado et al. (2010)	Experimental	MU
*Allium sativum* L.	Amaryllidaceae	Mashour et al. (1998); Li et al. (2015)	Experimental	ACEI, ND, TL, AA
*Crinum glaucum* A.Chev	Amaryllidaceae	Tabassum and Ahmad (2011)	Experimental	MU
*Rhus chinensis* Mill.	Anacardiaceae	Anwar et al. (2016)	Experimental	AB, ND
*Annona muricata* L.	Annonaceae	Tabassum and Ahmad (2011)	Experimental	MU
*Ammi visnaga* (L.) Lam.	Apiaceae	Khan et al. (2001); Rauwald et al. (1994); Bhagavathula et al. (2015)	Experimental	CCB, DIU
*Angelica dahurica* (Hoffm.) Benth. & Hook.f. ex Franch. & Sav.	Apiaceae	Sarker and Nahar (2004); Lee, Shin, et al. (2015); Lu et al. (2016)	Experimental	CCB, DIU
*Apium graveolens* L.	Apiaceae	Tsi et al. (1995); Moghadam et al. (2013)	Experimental	DIU
*Coriandrum sativum* L.	Apiaceae	Jabeen et al. (2009); Dhanapakiam et al. (2008)	Experimental	CCB, DIU
*Daucus carota* L.	Apiaceae	Gilani et al. (2000); Nicolle et al. (2003)	Experimental	CCB
*Ligusticum wallichii* Franch	Apiaceae	Mashour et al. (1998)	Experimental	CCB, AB
*Petroselinum crispum* (Mill.) Fuss	Apiaceae	Kreydiyyeh and Usta (2002); Farzaei et al. (2013); El Rabey et al. (2017)	Experimental	DIU
*Alstonia scholaris* (L.) R. Br.	Apocynaceae	Bello et al. (2015)	Experimental	CCB
*Apocynum venetum* L.	Apocynaceae	Xie et al. (2012)	Experimental	DIU, ND, PDEI
*Aspidosperma subincanum* Mart. ex A.DC.	Apocynaceae	Bernardes et al. (2013)	Experimental	CCB, PDEI
*Rauvolfia serpentina* (L.) Benth. ex Kurz	Apocynaceae	Klabunde (2012)	Experimental	AB
*Panax ginseng* C.A.Mey.	Araliaceae	Liu and Huang (2016)	Experimental	CCB
*Ruscus aculeatus* L.	Asparagaceae	Mashour et al. (1998)	Experimental	AB, CCB
*Calendula officinalis* L.	Asteraceae	Calvo and Cavero (2014)	Traditional	DIU
*Chamaemelum nobile* (L.) All.	Asteraceae	Calvo and Cavero (2014)	Traditional	MU
*Cynara cardunculus* L.	Asteraceae	Waltenberger et al. (2016)	Experimental	AA
*Dendranthema morifolium* (Ramat.) Tzvelev	Asteraceae	Jin et al. (2016)	Experimental	CCB
*Gynura procumbens* (Lour.) Merr.	Asteraceae	Hoe et al. (2007)	Experimental	ACEI
*Matricaria chamomilla* L.	Asteraceae	Baharvand-Ahmadi et al. (2016)	Traditional	MU
*Santolina chamaecyparissus* L.	Asteraceae	Calvo and Cavero (2014)	Traditional	DIU
*Silybum marianum* (L.) Gaertn	Asteraceae	Liperoti et al. (2017)	Experimental	MU
*Taraxacum campylodes* G.E.Haglund	Asteraceae	Calvo and Cavero (2014)	Traditional	DIU
*Berberis vulgaris* L.	Berberidaceae	Abushouk et al. (2017)	Experimental	ACEI, CAS, ND, PCB, TL
*Borago officinalis* L.	Boraginaceae	Baharvand-Ahmadi et al. (2016)	Traditional	MU
*Brassica napus* L.	Brassicaceae	Akbari et al. (2016); Quinn et al. (2017)	Experimental	ACEI
*Erysimum cheiranthoides* L.	Brassicaceae	Shan et al. (2001)	Experimental	DIU
*Lepidium latifolium* L.	Brassicaceae	Tabassum and Ahmad (2011)	Experimental	DIU
*Nasturtium officinale* R.Br.	Brassicaceae	Calvo and Cavero (2014)	Traditional	MU
*Raphanus raphanistrum *subsp.* sativus* (L.) Domin	Brassicaceae	Vargas et al. (1999); Lugasi et al. (2005); Ghayur and Gilani (2006)	Experimental	DIU, ND
*Commiphora wightii* (Arn.) Bhandari	Burseraceae	Rastogi et al. (2016)	Traditional	MU
*Dipterygium glaucum* Decne	Capparaceae	Ahmeda et al. (2015)	Traditional	MU
*Valeriana officinalis* L.	Caprifoliaceae	Baharvand-Ahmadi et al. (2016)	Traditional	MU
*Terminalia arjuna* (Roxb. ex DC.) Wight & Arn	Combretaceae	Rastogi et al. (2016)	Traditional	MU
*Commelina virginica* L.	Commelinaceae	Tabassum and Ahmad (2011)	Experimental	AB
*Cuscuta reflexa* Roxb.	Convolvulaceae	Gilani et al. (1992)	Experimental	CAS
*Bryophyllum pinnatum* (Lam.) Oken	Crassulaceae	Anwar et al. (2016)	Experimental	AA
*Sedum roseum* (L.) Scop.	Crassulaceae	Waltenberger et al. (2016)	Experimental	MU
*Momordica charantia* L.	Cucurbitaceae	Ojewole et al. (2006)	Experimental	MU
*Mukia maderaspatana* (L.) M.Roem.	Cucurbitaceae	Anwar et al. (2016)	Experimental	AA, TL
*Sechium edule* (Jacq.) Sw.	Cucurbitaceae	Ibarra-Alvarado et al. (2010)	Traditional	MU
*Elaeagnus rhamnoides* (L.) A. Nelson	Elaeagnaceae	Liu and Huang (2016)	Experimental	CCB
*Equisetum arvense* L.	Equisetaceae	Calvo and Cavero (2014)	Traditional	DIU
*Calluna vulgaris* (L.) Hull	Ericaceae	Calvo and Cavero (2014)	Traditional	DIU
*Eucommia ulmoides* Oliv.	Eucommiaceae	Hosoo et al. (2015)	Experimental	ND
*Astragalus propinquus* Schischkin	Fabaceae	Zhang et al. (1997); Liu and Huang (2016)	Experimental	AA, TL
*Castanospermum australe* A.Cunn. & C. Fraser	Fabaceae	Tabassum and Ahmad (2011)	Experimental	MU
*Desmodium styracifolium* (Osbeck) Merr.	Fabaceae	Tabassum and Ahmad (2011); Chen et al. (2015)	Experimental	AB, TL
*Glycine max* (L.) Merr.	Fabaceae	Lee et al. (2017)	Experimental	AA
*Medicago sativa* L.	Fabaceae	Bora and Sharma (2011)	Experimental	AA, TL
*Melilotus officinalis* (L.) Pall.	Fabaceae	Pirmohamed (2006)	Experimental	TL
*Pueraria montana var. lobata* (Willd.) Sanjappa & Pradeep	Fabaceae	Tabassum and Ahmad (2011); Chen et al. (2015)	Experimental	AB, TL
*Senna occidentalis* (L.) Link	Fabaceae	Anwar et al. (2016)	Experimental	CCB
*Styphnolobium japonicum* (L.) Schott	Fabaceae	Chen and Hsieh (2010)	Experimental	TL
*Trigonella foenum-graecum* L.	Fabaceae	Taj Eldin et al. (2013)	Experimental	TL
*Centaurium erythraea* Rafn	Gentianaceae	Calvo and Cavero (2014)	Traditional	MU
*Halenia elliptica* D. Don	Gentianaceae	Li et al. (2015)	Experimental	CCB
*Ribes divaricatum* Douglas	Grossulariaceae	Baharvand-Ahmadi et al. (2016)	Traditional	MU
*Hypericum perforatum* L.	Hypericaceae	Baharvand-Ahmadi et al. (2016)	Traditional	MU
*Crocus sativus* L.	Iridaceae	Joukar and Dehesh (2015)	Experimental	CAS
*Coleus forskohlii* (Willd.) Briq.	Lamiaceae	Christenson et al. (1995); Loftus et al. (2015)	Experimental	AA, TL
*Dracocephalum moldavica* L.	Lamiaceae	Ebrahim Sajjadi et al. (1998); Miernisha et al. (2016)	Experimental	ND, TL
*Lavandula stoechas* L.	Lamiaceae	Gilani et al. (2000)	Experimental	CCB
*Leonurus cardiaca* L.	Lamiaceae	Zou et al. (1989)	Experimental	TL
*Mentha suaveolens* Ehrh	Lamiaceae	Bello et al. (2001)	Experimental	AB
*Ocimum basilicum* L.	Lamiaceae	Amrani et al. (2009); Umar et al. (2010)	Experimental	ACEI, ERA, TL
*Rosmarinus officinalis* L.	Lamiaceae	Ulbricht et al. (2010); Ibarra et al. (2011)	Experimental	ACEI, TL
*Salvia miltiorrhiza* Bunge	Lamiaceae	Ling et al. (2008); Liu and Huang (2016)	Experimental	TL
*Scutellaria baicalensis* Georgi	Lamiaceae	Króliczewska et al. (2011); Lee, Ku et al. (2015); Liu and Huang (2016)	Experimental	ND, TL
*Cinnamomum verum* J.Presl	Lauraceae	Anwar et al. (2016)	Experimental	ND
*Linum usitatissimum* L.	Linaceae	Tabassum and Ahmad (2011)	Experimental	AA, TL
*Punica granatum* L.	Lythraceae	Tabassum and Ahmad (2011)	Experimental	ACEI
*Magnolia grandiflora* L.	Magnoliaceae	Ibarra-Alvarado et al. (2010)	Experimental	MU
*Abelmoschus manihot* (L.) Medik.	Malvaceae	Lv et al. (2017)	Experimental	AA
*Abroma augusta* (L.) L.f.	Malvaceae	Khanra et al. (2015)	Experimental	AA
*Gossypium barbadense* L.	Malvaceae	Hasrat et al. (2004)	Experimental	MU
*Hibiscus sabdariffa* L.	Malvaceae	Chen et al. (2003); Ojeda et al. (2010)	Experimental	AA, ACEI
*Theobroma cacao* L.	Malvaceae	Osakabe and Yamagishi (2009); Tabassum and Ahmad (2011); Sarriá et al. (2012)	Experimental	AA, ND
*Veratrum album* L.	Melanthiaceae	Swiss and Maison (1952); Mashour et al. (1998)	Experimental	CAS
*Stephania tetrandra* S. Moore	Menispermaceae	Mashour et al. (1998)	Experimental	CCB
*Artocarpus altilis* (Parkinson ex F.A. Zorn) Fosberg	Moraceae	Tabassum and Ahmad (2011)	Experimental	AB
*Ficus religiosa* L.	Moraceae	Baharvand-Ahmadi et al. (2016)	Traditional	MU
*Morus alba* L.	Moraceae	Kim et al. (2017)	Experimental	TL
*Peganum harmala* L.	Nitrariaceae	Gilani et al. (1992)	Experimental	MU
*Fraxinus angustifolia* Vahl	Oleaceae	Calvo and Cavero (2014)	Traditional	DIU
*Olea europaea* L.	Oleaceae	Micucci et al. (2015)	Experimental	AA, CCB
*Fuchsia magellanica* Lam.	Onagraceae	Tabassum and Ahmad (2011)	Traditional	DIU
*Cistanche tubulosa* (Schenk) Wight	Orobanchaceae	Li et al. (2015)	Experimental	ND
*Bocconia frutescens* L.	Papaveraceae	Ibarra-Alvarado et al. (2010)	Experimental	MU
*Chelidonium majus* L.	Papaveraceae	Calvo and Cavero (2014)	Traditional	DIU
*Sesamum indicum* L.	Pedaliaceae	Anwar et al. (2016)	Experimental	AA, ND
*Phyllanthus amarus* Schumach. & Thonn.	Phyllanthaceae	Anwar et al. (2016)	Experimental	AA, DIU
*Pinus pinaster* Aiton	Pinaceae	Tabassum and Ahmad (2011)	Experimental	ACEI
*Digitalis purpurea* L.	Plantaginaceae	Mashour et al. (1998)	Experimental	CG
*Avena sativa* L.	Poaceae	Anwar et al. (2016)	Experimental	AA, ND
*Hordeum vulgare* L.	Poaceae	d’Avigdor et al. (2014)	Traditional	MU
*Reynoutria multiflora* (Thunb.) Moldenke	Polygonaceae	Liu and Huang (2016)	Experimental	AA
*Rheum palmatum* L.	Polygonaceae	Hamzeh et al. (2014)	Experimental	AA
*Rumex abyssinicus* Jacq.	Polygonaceae	d’Avigdor et al. (2014)	Traditional	MU
*Embelia ribes* Burm.f.	Primulaceae	Anwar et al. (2016)	Experimental	AA
*Coptis chinensis* Franch.	Ranunculaceae	Al Disi et al. (2015)	Experimental	CCB, ND, TL
*Nigella sativa* L.	Ranunculaceae	Jaarin et al. (2015)	Experimental	AA, ACEI, ND
*Rhamnus alaternus* L.	Rhamnaceae	Calvo and Cavero (2014)	Traditional	MU
*Ziziphus jujuba* Mill.	Rhamnaceae	Baharvand-Ahmadi et al. (2016)	Traditional	AA
*Crataegus pinnatifida* Bunge	Rosaceae	Tabassum and Ahmad (2011); Wang et al. (2013)	Experimental	AA, ND
*Filipendula ulmaria* (L.) Maxim.	Rosaceae	Jerie (2006); Calvo and Cavero (2014)	Experimental	AA, DIU, TL
*Malus sylvestris* (L.) Mill.	Rosaceae	Calvo and Cavero (2014)	Traditional	MU
*Potentilla reptans* L.	Rosaceae	Calvo and Cavero (2014)	Traditional	MU
*Prunus spinosa* L.	Rosaceae	Calvo and Cavero (2014); Marchelak et al. (2017)	Experimental	AA, DIU
*Tetradium ruticarpum* (A.Juss.) T.G.Hartley	Rutaceae	Mashour et al. (1998)	Experimental	ND
*Salix alba* L.	Salicaceae	Mahdi (2010)	Experimental	AA, TL
*Viscum album* L.	Santalaceae	Baharvand-Ahmadi et al. (2016)	Traditional	MU
*Aesculus hippocastanum* L.	Sapindaceae	Mashour et al. (1998); Calvo and Cavero (2014)	Experimental	VENO
*Schisandra chinensis* (Turcz.) Baill.	Schisandraceae	Kim et al. (2017)	Experimental	TL
*Verbascum sinuatum* L.	Scrophulariaceae	Calvo and Cavero (2014)	Traditional	MU
*Atropa belladona* L.	Solanaceae	Davies and Hollman (2002)	Experimental	MRA
*Lycium barbarum* L.	Solanaceae	Zhang et al. (2015)	Experimental	ND
*Physalis alkekengi* L.	Solanaceae	Baharvand-Ahmadi et al. (2016)	Traditional	MU
*Camellia sinensis* (L.) Kuntze	Theaceae	Nantz et al. (2009)	Experimental	AA
*Tropaeolum majus* L.	Tropaeolaceae	Anwar et al. (2016)	Experimental	ACEI, DIU, ND
*Cecropia pachystachya* Trécul	Uritaceae	Liu and Huang (2016)	Experimental	CG
*Musanga cecropioides* R.Br. ex Tedlie	Urticaceae	Adeneye et al. (2006)	Experimental	ACEI
*Parietaria judaica* L.	Urticaceae	Calvo and Cavero (2014)	Traditional	MU
*Urtica dioica* L.	Urticaceae	Calvo and Cavero (2014)	Traditional	MU
*Viola odorata* L.	Violaceae	Anwar et al. (2016)	Experimental	AA, CCB, ND
*Vitis vinifera* L.	Vitaceae	Dohadwala and Vita (2009)	Experimental	AA, TL
*Alpinia zerumbet* (Pers.) B.L.Burtt & R.M.Sm.	Zingiberaceae	Lin et al. (2008); da Cunha et al. (2013)	Experimental	CCB
*Elettaria cardamomum* (L.) Maton	Zingiberaceae	Anwar et al. (2016); Nagashree et al. (2017)	Experimental	CCB
*Kaempferia parviflora* Wall. ex Baker	Zingiberaceae	Achuthan and Padikkala (1997); Anwar et al. (2016)	Experimental	CCB, ND
*Zingiber officinale* Roscoe	Zingiberaceae	Ghayur and Gilani (2005); Bhandari et al. (2005)	Experimental	CCB
*Tribulus terrestris* L.	Zygophyllaceae	Anwar et al. (2016)	Experimental	ACEI, ND

Scientific names follow the latest taxonomic changes in PlantList (http://www.theplantlist.org/), and may differ from the name used in the reference. For each species, the specific traditional application or pharmacological mechanism as described in the reference is indicated. Pharmacological mechanisms were classified according to Klabunde (2012). These mechanisms included angiotensin-converting enzyme inhibitors (ACEI), alpha/beta-adrenoceptor blockers (AB), calcium-channel blockers (CCB), cardiac glycosides or Na+/K + ATPase inhibitors (CG), diuretics (DIU), nitrodilators (ND), phosphodiesterase-inhibitors (PDEI), potassium channel blockers (PCB), thrombolytics (TL), endothelium-receptor antagonist (ERA), muscarinic receptor antagonist (MRA), venotonic (VENO) and anti-atherosclerosis (AA). MU was indicated when the mechanism is unknown.

## Results

**Figure 1.**
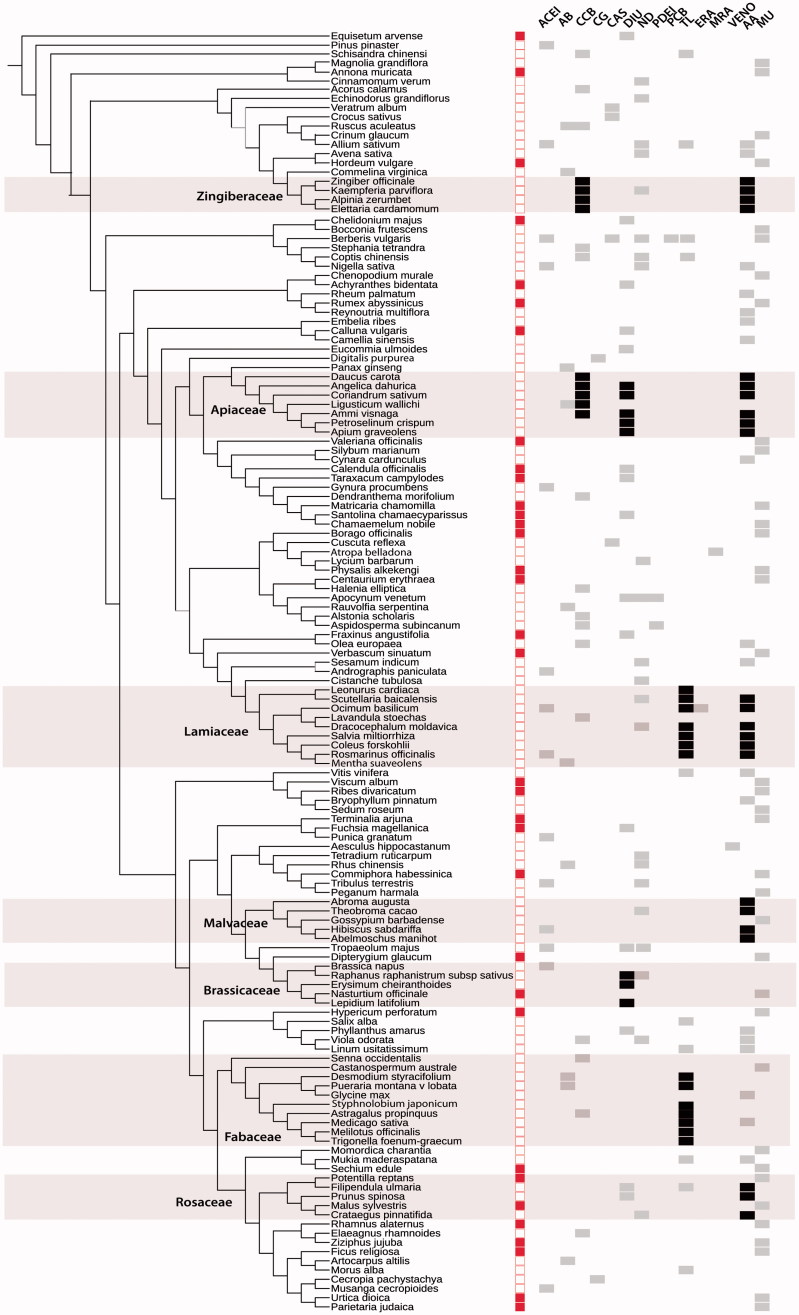
Phylogeny of 139 plant species with cardiovascular applications and their pharmacological mechanisms of actions (black and grey boxes). Plant families with 4 or more species, possessing common pharmacological mechanisms of action (black boxes) are highlighted and labeled. Mechanisms included angiotensin-converting enzyme inhibitors (ACEI), alpha/beta-adrenoceptor blockers (AB), calcium-channel blockers (CCB), cardiac glycosides or Na+/K+ ATPase inhibitors (CG), diuretics (DIU), nitrodilators (ND), phosphodiesterase-inhibitors (PDEI), potassium channel blockers (PCB), anticoagulant/thrombolytics (TL), endothelium-receptor antagonist (ERA), muscarinic receptor antagonist (MRA), venotonic (VENO), and anti-atherosclerosis (AA). MU was indicated when the mechanism is unknown. Red solid boxes immediately next to species name indicate that the plant has only ethnobotanical/traditional use.

The plant phylogeny shows that out of the 139 species from 71 plant families (Table 1), seven families with 45 species (Apiaceae, Brassicaceae, Fabaceae, Lamiaceae, Malvaceae, Rosaceae and Zingiberaceae) had disproportionately more species (at least 4) relative to other families and revealed common pharmacological mechanisms of action (black boxes), which are discussed below (Figure 1). Families with four or more species demonstrating the same mechanism of action are considered pharmacologically important for CV drug development.

Five of seven species in Apiaceae and all four species in Zingiberaceae demonstrated activity as calcium channel blockers (CCBs). Five of seven species in Apiaceae, and 3/5 species in Brassicaceae exhibited diuretic mechanism of action. Most Lamiaceae (7/9) and Fabaceae (7/10) members showed anticoagulant/thrombolytic activity. Moreover, most species of Apiaceae (6/7), Lamiaceae (6/9), Malvaceae (4/5), Rosaceae (3/5) and Zingiberaceae (4/4) were found to have anti-atherosclerosis effects (e.g., cholesterol/lipid-lowering properties). Some plant species only have ethnobotanical evidence (indicated by the red box next to species name in Figure 1), and their precise mechanism of action is yet undetermined. However, some of these species are confamilial with species that have a predominant pharmacological mechanism of action, thus, it may be deduced that these species would contain similar phytochemistry and potentially exert the same mechanism.

## Discussion

The reconstructed phylogeny of CV plants (Figure 1) conforms to the expected phylogenetic relationships by the Angiosperm Phylogeny Group (APG 2016). Seven plant families had disproportionately more species and possessed common pharmacological mechanisms of action based on experimental evidence (Table 1). These were Apiaceae, Brassicaceae, Fabaceae, Lamiaceae, Malvaceae, Rosaceae and Zingiberaceae, which collectively exhibited calcium-channel blocking activity, anticoagulant/thrombolytic and diuretic effects, as well as anti-atherosclerosis properties (Figure 1). It may be argued that the identification of these families in the CV phylogeny may be an artifact of their increased biodiversity. This may be true for Fabaceae, which is among the most speciose families. However, Orchidaceae, Asteraceae, Rubiaceae and Poaceae are also among these mega-diverse families (Christenhusz and Byng 2016), but interestingly, these families did not appear as being overrepresented in the CV phylogeny. Thus, families highlighted in our study indeed possess evolutionarily important pharmacological properties that may be exploited for CV drug discovery.

### Cardiovascular pharmacological mechanisms of natural products

Aspirin, amiodarone, digoxin, reserpine, tetrandrine and warfarin are modern-day CV drugs developed from plant natural products (Mashour et al. 1998; Heinrich et al. 2012; Li et al. 2015), and their source species were included in this study. These drugs exert varying mechanisms of action. Some of the most commonly prescribed medications lower blood pressure, by either inhibiting angiotensin (ACE inhibitors), or by preventing epinephrine and norepinephrine from binding to adrenoceptors, consequently relaxing the heart and arterial pressure (α/β-blockers). Reserpine, depletes these catecholamines preventing them from binding to adrenoceptors (Klabunde 2012), and was used in the past to treat hypertension, but with the development of newer hypertensive drugs, it has become less favoured (Shamon and Perez 2009). Diuretics, on the other hand, lower blood pressure by removing salt and fluid from the body increasing urine output. Nitrodilators (e.g., nitroglycerin) produce nitric oxide (NO) within tissues mimicking endogenous NO, promoting vasodilation (Klabunde 2012). Statins are lipid-lowering drugs that inhibit a liver enzyme important in cholesterol synthesis, helping prevent atherosclerosis and hypertension (Milionis et al. 2006). Anticoagulants and thrombolytics, such as aspirin and warfarin, prevent or dissolve blood clots, which may form in coronary, cerebral or pulmonary arteries, where they can be immediately life-threatening (Klabunde 2012). Aspirin prevents platelet aggregation, while warfarin antagonizes vitamin K, limiting blood clotting (Almony et al. 1996). When hypertension presents with angina (chest pain) and/or arrhythmia (abnormal heart rate), CCBs may be prescribed (Ryan 1990), such as tetrandrine (Dai et al. 1990; Sutter and Wang 1993). CCB prevents influx of calcium into cardiac muscle cells, thus depressing heart activity and lowering blood pressure. Another anti-arrhythmia is digoxin, which is a cardiac glycoside that inhibits the Na^+^/K^+^-ATP pump, causing intracellular sodium and calcium concentrations to increase, thereby increasing heart contractility in cases of congestive heart failure (Klabunde 2012). Amiodarone is also another anti-arrhythmia but works as a PCB to prolong the period of time that the cell is unexcitable and is useful in suppressing tachycardia (Auer et al. 2002). Less common mechanisms found for plant species in this study were PDEIs, endothelium receptor antagonist (ERA), MRA and VENO agents. PDEI stimulates the heart but has vasodilatory effect; MRA antagonizes acetylcholine effects on the heart, and can reverse bradycardia (slow heart rate) (Klabunde 2012); and VENO agents increase venous tone and treat venous disorders like varicose veins (Mashour et al. 1998).

### Phytochemistry of Apiaceae and Zingiberaceae as calcium channel blockers

In Apiaceae, 5/7 species were found to possess CCB activity. The lipophilic extract of *Ammi visnaga* fruits contained the coumarin visnadin, which mediated this effect (Rauwald et al. 1994). The crude extract of coriander, *Coriandrum sativum*, was shown to have gut inhibitory activity via calcium antagonism (Jabeen et al. 2009). The methanol extract of *Angelica dahurica* induced vasorelaxation on rat aorta by blockade of calcium channels, perhaps due to its furocoumarin content (Lee, Shin, et al. 2015). The same mechanism was demonstrated for fractions of aerial parts of the common carrot, *Daucus carota*, containing coumarin glycosides (Gilani et al. 2000). Though not a coumarin derivative like in previously described species, tetramethylpyrazine, the active constituent in *Ligusticum wallichii*, was also found to mediate its hypotensive effect via CCB activity (Mashour et al. 1998).

All four species of the unrelated Zingiberaceae family demonstrated CCB effect. Thai black ginger, *Kaempferia parviflora*, when given to rats, had a vasorelaxant effect that was achieved by reducing Ca^2+^ influx due to its 5,7-dimethoxyflavone content (Anwar et al. 2016). This was also the case for the confamilial cardamom, *Elettaria cardamomum* (Anwar et al. 2016). The methanolic fraction of the essential oil of *Alpinia zerumbet*, folklorically used in Brazil to treat hypertension, was also shown to inhibit calcium influx promoting hypotension (da Cunha et al. 2013). Extracts of common ginger, *Zingiber officinale* also lowered blood pressure and possessed cardiodepressant activity via the same mechanism (Ghayur and Gilani 2005). The common mechanism of CCB among these species of Zingiberaceae invites investigation of other species within the family for this activity, as predicted by the phylogeny. As a proof of concept, when other species were searched for CCB effects, *Curcuma longa* (turmeric) came up positive due to its constituent molecule cyclocurcumin (Kim et al. 2017), as well as several other species within Zingiberaceae (Gonçalves et al. 2014). This reinforces the utility of the phylogeny in drug discovery, in predicting pharmacological mechanisms of novel and unexplored species based on their evolutionary relationships.

### Phytochemistry of Apiaceae and Brassicaceae as diuretics

Experimental studies have repeatedly shown that members of the family Apiaceae work as diuretics. Diuretics increase urine output and lower blood pressure by inhibiting the reabsorption of sodium at different parts of the renal tubular system (Klabunde 2012). *Ammi visnaga* has been used traditionally in Egypt to treat kidney stones (Vanachayangkul et al. 2010), and has been demonstrated to possess potent diuretic activity due to its bioactive component khellin (Khan et al. 2001; Günaydin and Beyazit 2004). The confamilial *Angelica dahurica*, an important medicinal plant in the Far East, has also been traditionally used as diuretic (Sarker and Nahar 2004). In celery, *Apium graveolens*, diuresis due to its constituent, *n*-butylphthalide, promoted its antihypertensive effect (Moghadam et al. 2013). The crude extract of coriander, *Coriandrum sativum*, similarly worked as a diuretic in an experiment in rats (Jabeen et al. 2009). In parsley, *Petroselinum crispum*, phenolic compounds, flavonoids and essential oil components are believed to be responsible for many of its pharmacological activities including its diuretic and antiplatelet activity (Farzaei et al. 2013). Though there were no experimental studies found to support *Daucus carota’s* (carrot) or *Ligusticim wallichii’s* use as a diuretic, it is predicted that based on this phylogenetic pattern for Apiaceae, that these species may also promote diuresis.

Three of five species in the unrelated family, Brassicaceae, also exhibited diuretic activity. *Lepidium latifolium*, traditionally used in the Canary Islands to treat renal lithiasis (kidney stones), exhibited hypotensive effect due to its diuretic action (Tabassum and Ahmad 2011). The Chinese herbal species, *Erysimum cheiranthoides*, was shown to increase urine volume and decrease potassium channel activity of the kidney, most likely due to its cardiotonic glycosides such as erysimin (Shan et al. 2001). *Raphanus sativus* (syn. *Raphanus raphanistrum *subsp.* sativus*), or radish, was also experimentally found to promote diuresis in rats (Vargas et al. 1999). Other species in the family were found to exhibit other mechanisms of action (Figure 1 and Table 1). *Brassica napus* was found to exhibit another mechanism, as inhibitors of angiotensin-converting enzymes. *Nasturtium officinale* (watercress) is traditionally used to treat anaemia in Navarra, Spain, but has no known mechanism of action (Calvo and Cavero 2014). Though the specific phytochemistry promoting diuresis is not clear, since 3/5 species in Brassicaceae were experimentally shown to be diuretic, we can predict that the other species may exert this action. This phylogenetic pharmacological pattern could guide future research of plant species that have yet to be experimentally studied, such as *N. officinale.*

### Phytochemistry of Fabaceae and Lamiaceae as anticoagulant/thrombolytic agents

Seven out of 10 species from the Fabaceae family have shown a TL effect, which prevents platelet aggregation or dissolves a blood clot (Klabunde 2012). The popular blood thinner Warfarin was developed from the anticoagulant dicoumarol found in moldy sweet clover, *Melilotus officinalis*, which is a member of Fabaceae (Pirmohamed 2006). Interestingly, other species in the family were also found to be TL. The saponin astragaloside in *Astragalus propinquus* can increase the fibrinolytic potential of cultured human umbilical vein endothelial cells (Zhang et al. 1997). Chen et al. (2015) reported that *Pueraria lobata* (syn. *Pueraria montana var. lobata*) and *Desmodium styracifolium* showed fibrinolytic activity, with that of *D. styracifolium* similar to that of the positive drug urokinase. The isoflavone puerarin from the species *P. thomsonii* and *P. lobata* has been found to reduce blood viscosity, promote cerebral blood flow and reduce red blood cell aggregation and secondary cerebral thrombosis (Yuan et al. 2017). Certain isoflavones in S*ophora japonica* (syn. *Styphnolobium japonicum*) were strong inhibitors of arachidonic acid- and thromboxane A_2_-induced platelet aggregation in rat plasma (Chen and Hsieh 2010). Aqueous extracts of fenugreek, *Trigonella foenum-graecum*, inhibited the coagulation process *in vitro* and significantly prolonged prothrombin time in a dose-dependent manner (Taj Eldin et al. 2013). A review paper on alfalfa, *Medicago sativa*, described it as beneficial for blood clotting disorders, and may be contraindicated in those who take blood thinners (Bora and Sharma 2011). It seems that the common occurrence of flavonoids, such as isoflavones and coumarins in Fabaceae is responsible for this pharmacological pattern. Though other species were not found to share this pattern (*Castanospermum australe*, *Senna occidentalis* and *Glycine max*), we can speculate that further research and testing may yield phytochemicals with this activity.

The unrelated Lamiaceae family has also been shown to be TL for 7/9 species. *Dracocephalum moldavica*, traditionally used in Uyghur (Turkish) medicine, possessed polyphenolics, rosmarinic acid and tilianin, that inhibited platelet aggregation of plasma (Miernisha et al. 2016). Rosmarinic acid was first isolated from rosemary, *Rosmarinus officinalis* (Shekarchi et al. 2012), and may also be responsible for this species’ antithrombotic activity (Ulbricht et al. 2010). In traditional Chinese medicine, *Salvia miltiorrhizae* (danshen) is used to treat CVDs, and was also shown to inhibit platelet adhesion and aggregation, as well as protect against myocardial ischemia, effects attributable to its phenolic components, salvianolic acid A and B and danshensu (salianic acid A) (Liu and Huang 2016). Polyphenolic-rich aqueous extracts of basil, *Ocimum basilicum*, also inhibited platelet aggregation (Amrani et al. 2009). The flavonoid baicalin in the Chinese herb, *Scutellaria baicalensis* was also TL (Lee, Ku, et al. 2015) as well as anti-inflammatory, and in fact, used in flavocoxid, a medical food product prescribed for osteoarthritis (Levy et al. 2010). In *Coleus forskohlii*, the diterpene forskolin was responsible for its anticoagulant effect (Christenson et al. 1995). Though the specific phytochemical was not identified in motherwort, *Leonurus cardiaca*, it was also demonstrated to inhibit blood clotting. Presence of polyphenols, in general, seems to have inhibitory effect on platelet aggregation (Nardini et al. 2007). Though other species within Lamiaceae, such as *Lavandula stoechas* and *Mentha spicata* demonstrated other mechanisms of action (Table 1; Figure 1), it is highly likely that these species, as well as other members of Lamiaceae have TL effects given the pharmacological patterns observed.

### Phytochemistry of plant families with anti-atherosclerosis potential

Most species of Apiaceae, Lamiaceae, Malvaceae, Rosaceae, Zingiberaceae in this study were found to have anti-atherosclerotic (AA) activity, either due to their antioxidant, anti-inflammatory, and/or lipid/cholesterol lowering abilities (Table 1 and Figure 1). Atherosclerosis is an inflammatory disease involving the accumulation of fat, cholesterol, calcium and other substances in the blood, resulting in the narrowing and hardening of the arteries (NHLBI 2015). Oxidation of low-density lipoprotein (LDL) is implicated in this inflammatory response, and dietary antioxidants, such as polyphenols and terpenoids (Grassmann 2005) have been shown to prevent LDL oxidation, and consequently, atherosclerosis progression (Fecka and Turek 2008; Fiedor and Burda 2014; Momiyama et al. 2014; Amarowicz and Pegg 2017). The abundance of polyphenols (flavonoids, coumarins, etc.) as well as terpenoids (e.g., carotenoids, monoterpenes, diterpenes) in species of Apiaceae (Pandey et al. 2012), Lamiaceae (Capecka et al. 2005), Malvaceae (De Oliveira et al. 2012), Rosaceae (Halvorsen et al. 2002) and Zingiberaceae (Habsah et al. 2000) most likely contributes to their AA effects (see also references in Table 1). Apiaceae and Malvaceae were previously identified by Xavier and Molina (2016) as potential sources of CV natural products from a phylogenetic analysis of culturally diverse herbal species used by immigrant populations in New York City. Our current study confirms the potential of these families as cardioprotective agents. Species from these strongly antioxidant families may then be explored as natural sources of lipid-lowering drugs, as an alternative and/or auxiliary therapy to prescription statins.

### Evolutionary pharmacology: the phylogeny as a predictive tool for cardiovascular drug discovery

Fabricant and Farnsworth (2001) posed the question, ‘What is the best approach to discover plants that contain potential drugs?’ They recommended that focusing on ethnomedicinally important plants is a good starting point and has been more successful in yielding new drug leads than the strategy of random plant collection. We have taken their word further, and analysed plant species with traditional and experimental evidence of CV application in a phylogenetic context to determine over-represented plant families. The families Apiaceae, Brassicaceae, Fabaceae, Lamiaceae, Malvaceae, Rosaceae and Zingiberaceae showed common pharmacological mechanisms of action for many species within their respective families, as expected, given the common ancestry. These evolutionary pharmacological patterns may be used to predict pharmacological traits in unexplored species within the group, particularly those that have only traditional evidence. This highlights the utility of the phylogeny in guiding drug discovery that has been exemplified in recent studies (Alrashedy and Molina 2016; Xavier and Molina 2016; Molina 2018).

Though there were only 139 species included in this study, this already allowed identification of seven families with CV importance. Sifting through additional ethnobotanical studies, we found that the same families were mentioned as being used for CV disorders by various cultures: Ayurvedic and Chinese (Jaiswal et al. 2016), Nigerian (Olorunnisola et al. 2015), Peruvian (De-la-Cruz et al. 2007), Slavic (Moskalenko 1987), Swiss (Abbet et al. 2014) and Turkish (Polat et al. 2013; Tetik et al. 2013). Asteraceae species were frequently cited in many studies, but we were unable to find a common pharmacological mechanism for its species, and its use in different cultures may be reflective of the incredible diversity of the family, thus easy accessibility. Other families, in addition to the seven we identified, have also been mentioned, but were unique to certain cultural groups, mostly likely due to their indigenous or localized distributions, such as tropical species from Combretaceae, Euphorbiaceae, Rutaceae, Meliaceae, or temperate species from Ericaceae and Betulaceae. Regardless, we anticipate that inclusion of more plant species and their mechanisms in the phylogeny would only serve to identify additional plant families that may be pharmacologically relevant.

## Conclusions

Phylogenetic and pharmacological analyses of plant species with CV applications have revealed plant families that have disproportionately more species relative to other families, with most species within the family exhibiting common mechanisms of action, as would be expected given the common ancestry. Evolutionary pharmacology, as applied in our study, directs us to these families and to their unexplored species, informing us of specific pharmacological assays to conduct given of what is known in experimentally tested related species, greatly expediting our search for new CV drugs.
